# Supplementary material for: Heme Oxygenase-1 Inhibition Modulates Autophagy and Augments Arsenic Trioxide Cytotoxicity in Pancreatic Cancer Cells
Source: Biomedicines. 2023 Sep 20;11(9):2580. doi: 10.3390/biomedicines11092580 (PMC10526552; doi:10.3390/biomedicines11092580)
Supplement: Supplementary file 1 [file biomedicines-11-02580-s001.zip › biomedicines-2585263-supplementary.pdf]

Figure S1

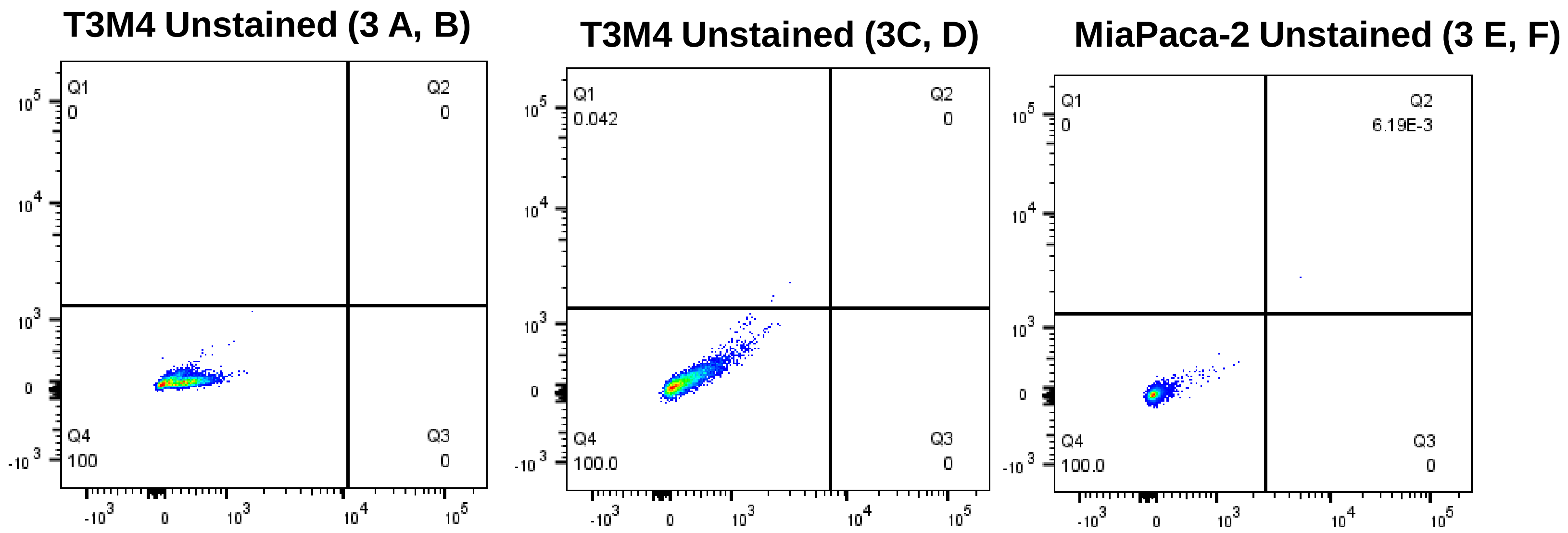
T3M4 Unstained (3 A, B)
Q1
0
Q2
0
Q4
100
Q3
0
T3M4 Unstained (3C, D)
Q1
0.042
Q2
0
Q4
100.0
Q3
0
MiaPaca-2 Unstained (3 E, F)
Q1
0
Q2
6.19E-3
Q4
100.0
Q3
0

Figure S2

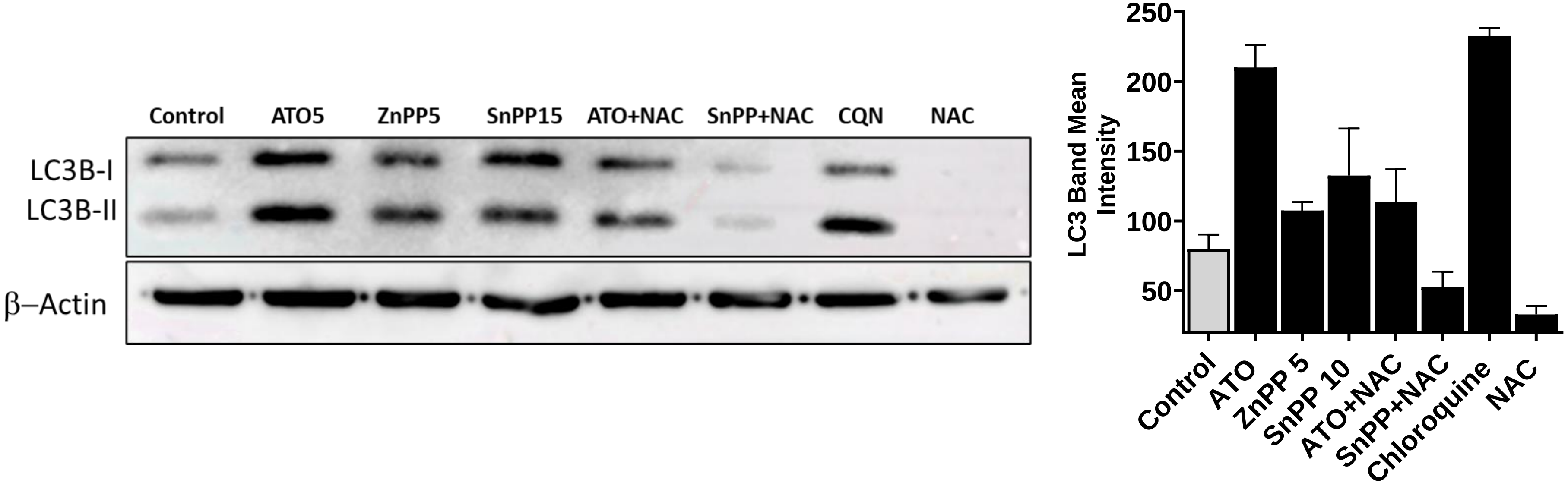
Control
ATO5
ZnPP5
SnPP15
ATO+NAC
SnPP+NAC
CQN
NAC
LC3B-I
LC3B-II
β–Actin
LC3 Band Mean Intensity
250
200
150
100
50
Control
ATO
ZnPP 5
SnPP 10
ATO+NAC
SnPP+NAC
Chloroquine
NAC

Figure S1. Unstained controls flow cytometry experiments in figure 3. Cells are not showing any auto-Fluorescence.

Figure S2 Inhibiting HO-1 suppress autophagy in ROS mediated mechanism. PDAC cells were treated with ATO (5 μM), SnPP (15 μM), NAC (1 mM), Chloroquine (15 μM) or combinations for 24 h. Control cells were treated with DMSO as a control. LC3 was measured by western blot. NAC reduced LC3 expression indicating that autophagy markers were increased by ROS mechanism. (Right) densitometric analysis of immunoblotting of autophagy protein LC3.
